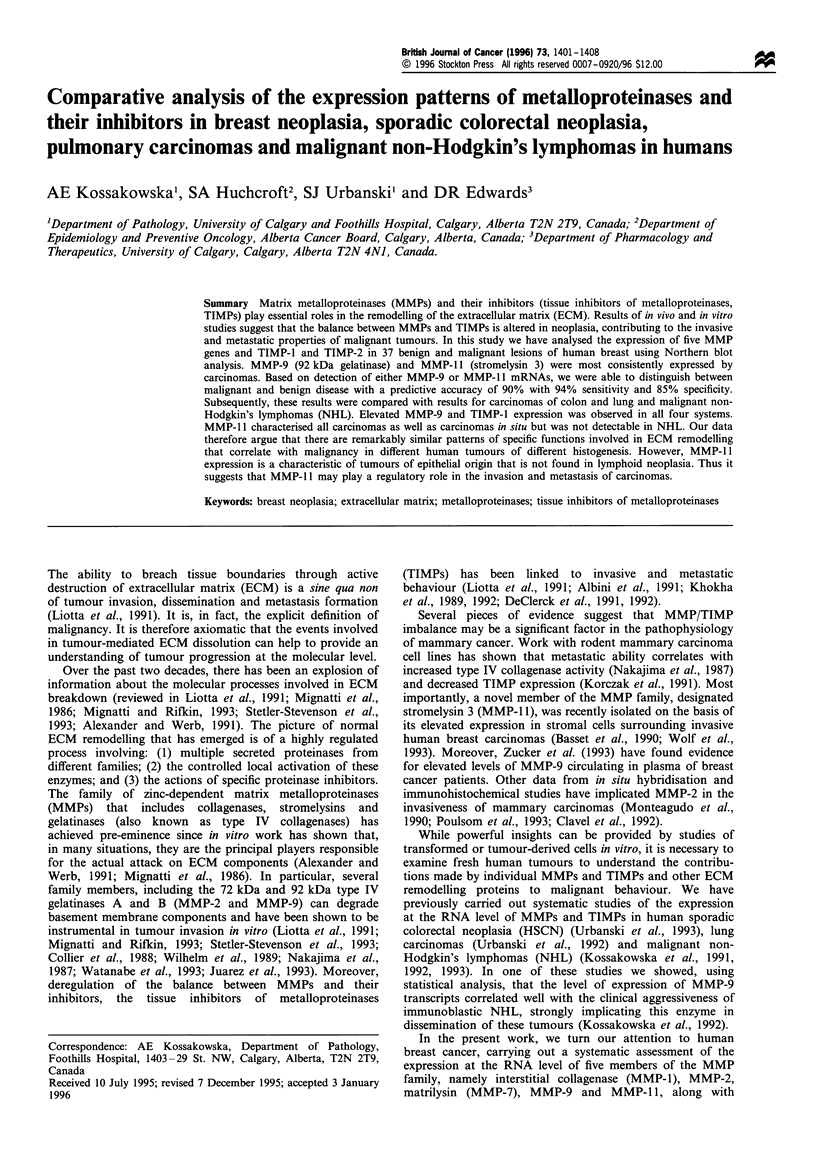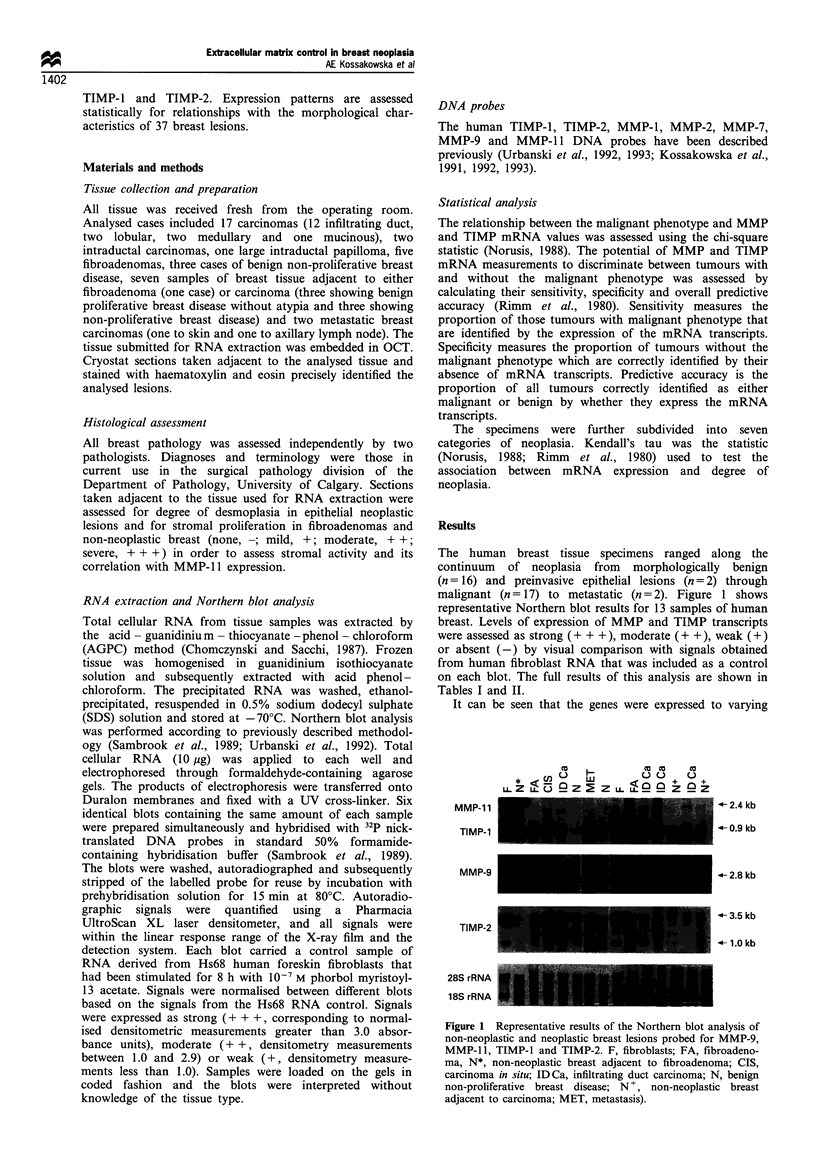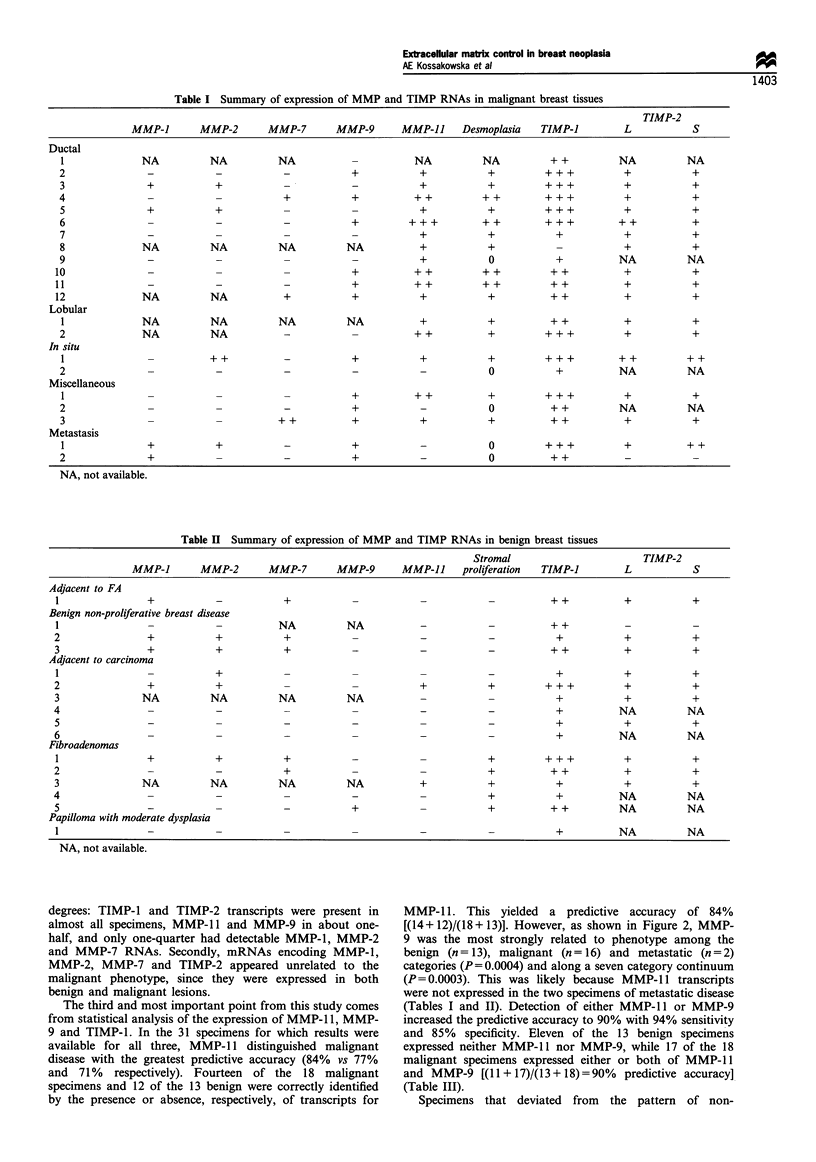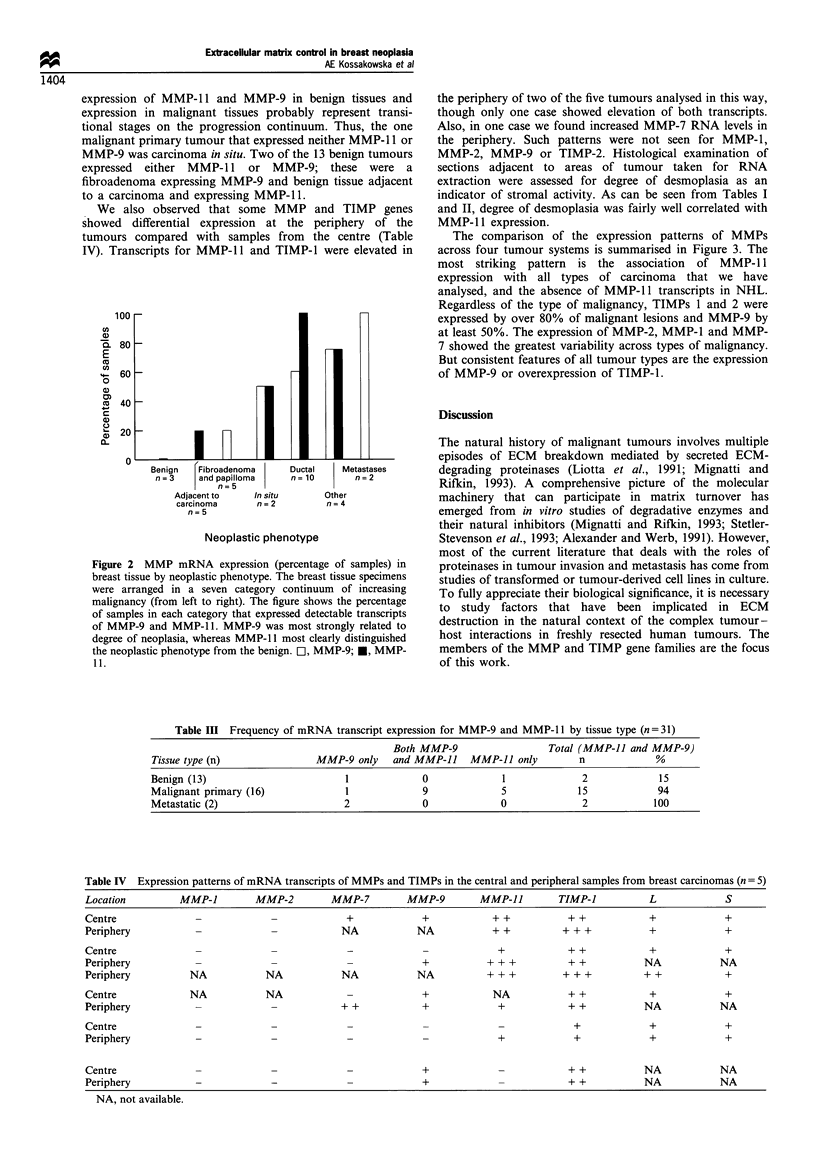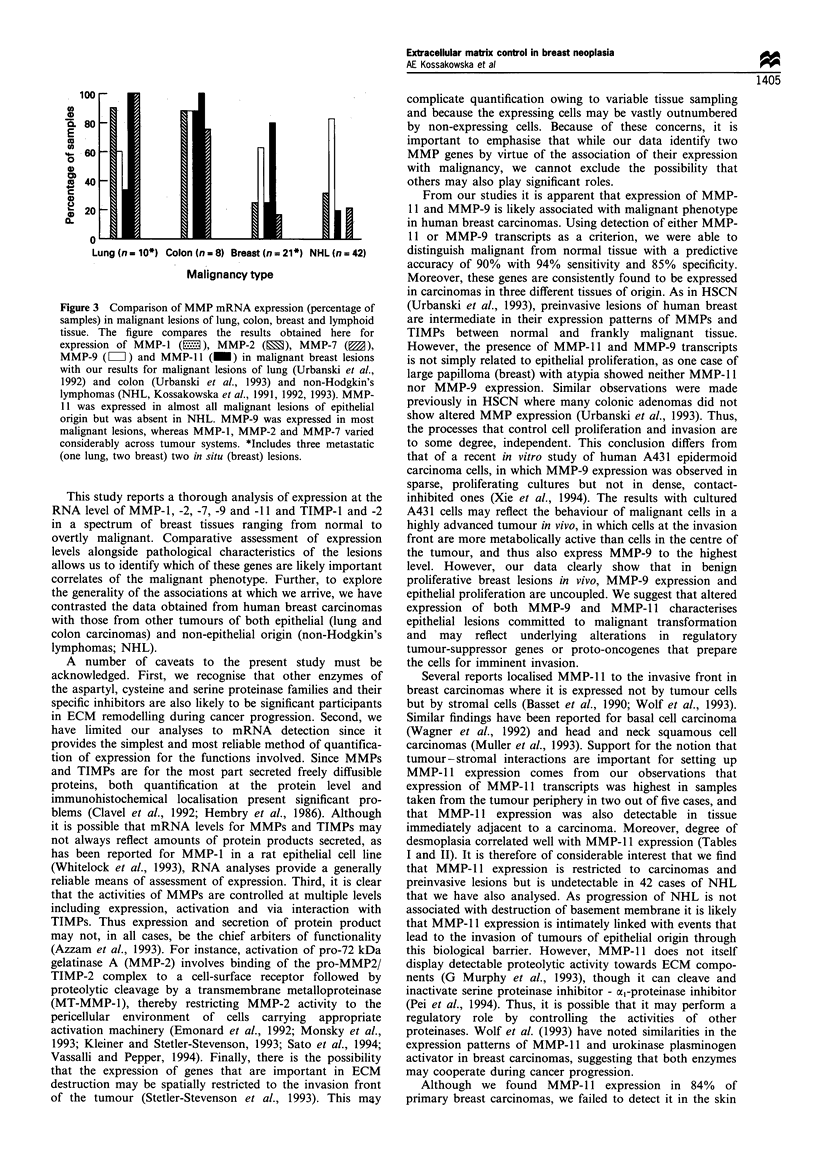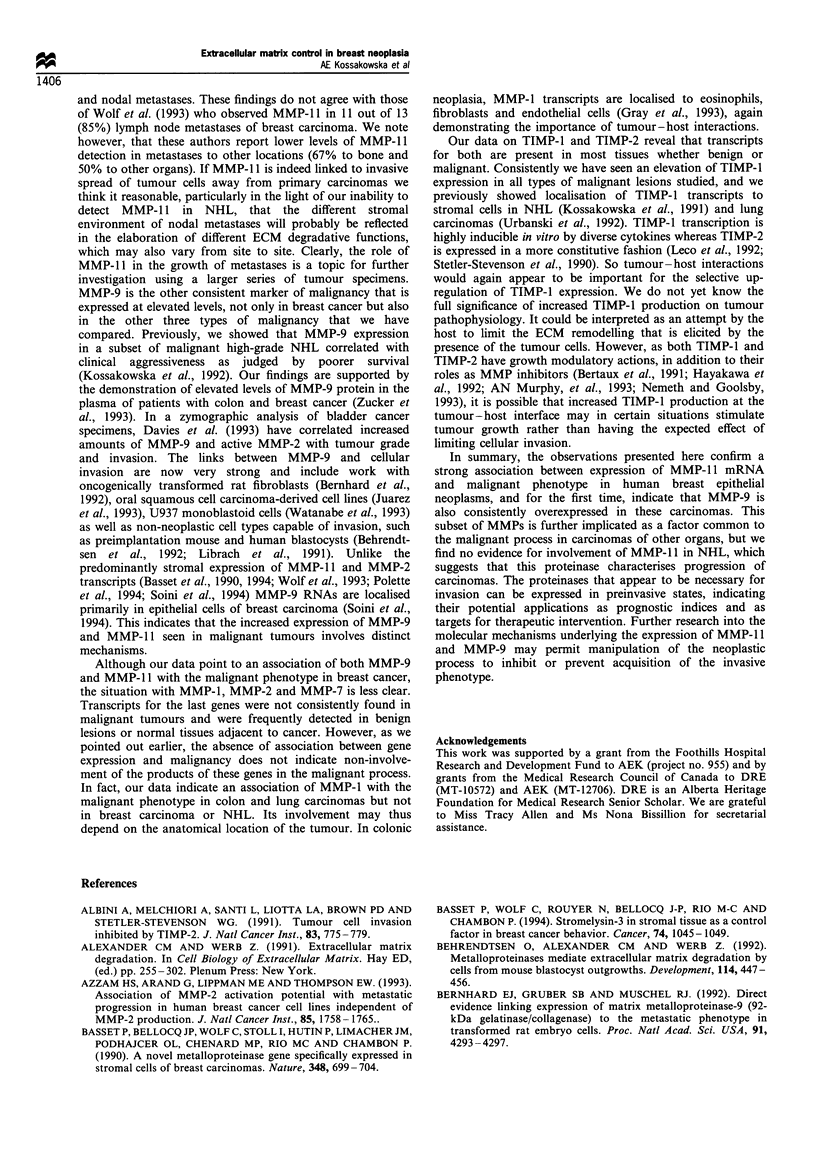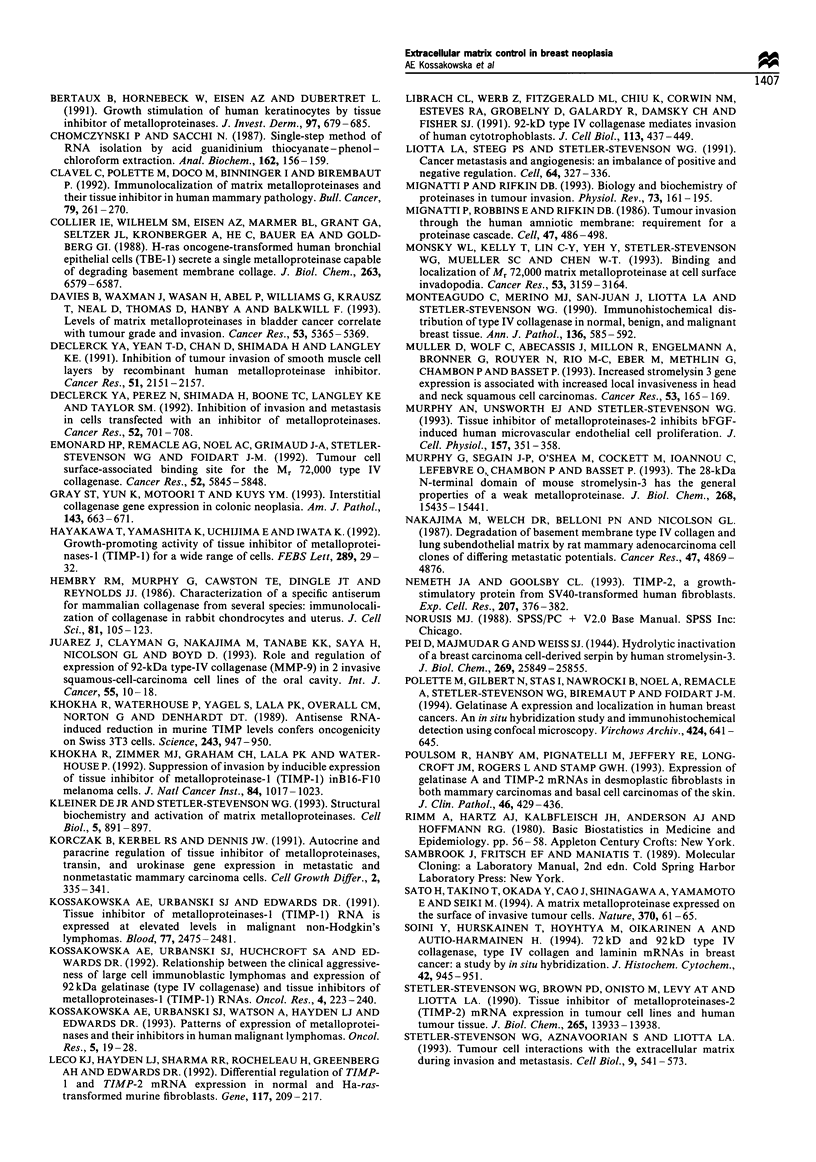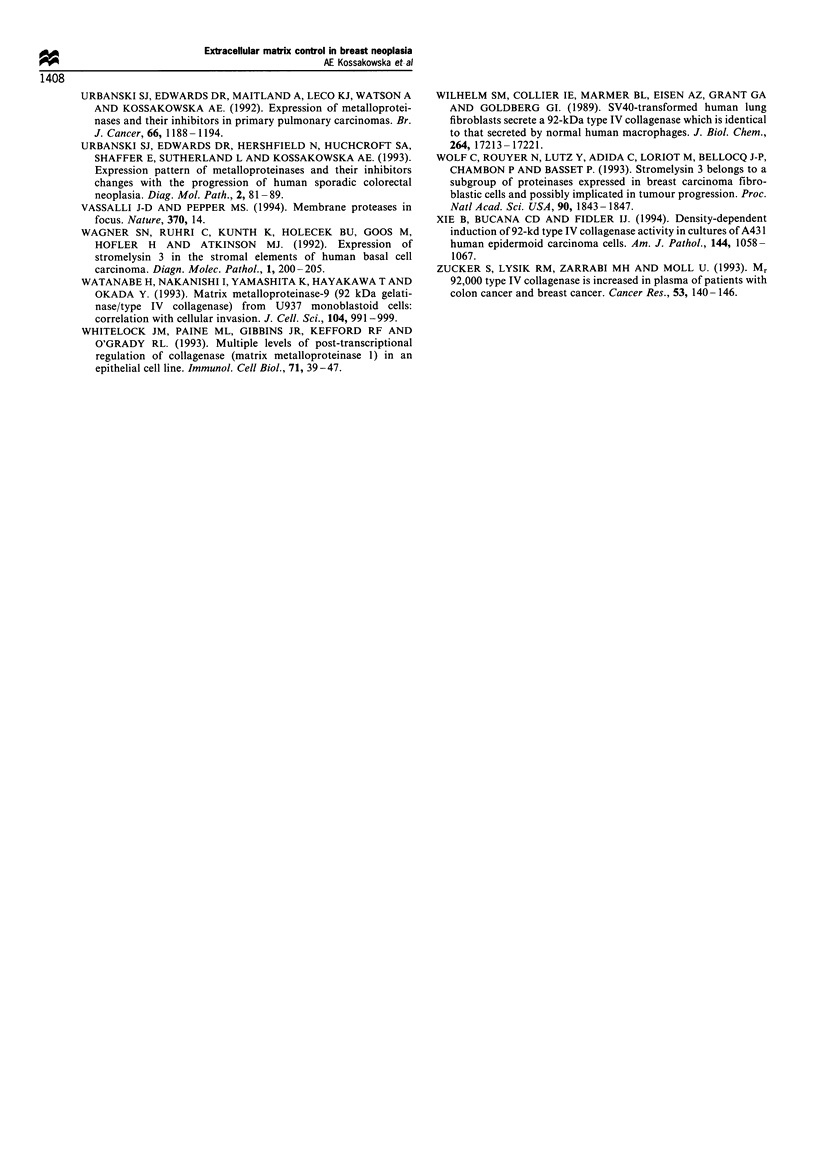# Comparative analysis of the expression patterns of metalloproteinases and their inhibitors in breast neoplasia, sporadic colorectal neoplasia, pulmonary carcinomas and malignant non-Hodgkin's lymphomas in humans.

**DOI:** 10.1038/bjc.1996.266

**Published:** 1996-06

**Authors:** A. E. Kossakowska, S. A. Huchcroft, S. J. Urbanski, D. R. Edwards

**Affiliations:** Department of Pathology, University of Calgary, Alberta, Canada.

## Abstract

**Images:**


British Journal of Cancer (1996) 73, 1401-1408
© 1996 Stockton Press All rights reserved 0007-0920/96 $12.00

### Comparative analysis of the expression patterns of metalloproteinases and their inhibitors in breast neoplasia, sporadic colorectal neoplasia, pulmonary carcinomas and malignant non-Hodgkin's lymphomas in humans

AE Kossakowska[1], SA Huchcroft[2], SJ Urbanski[1] and DR Edwards[3]

[1]*Department of Pathology, University of Calgary and Foothills Hospital, Calgary, Alberta T2N 2T9, Canada;* [2]*Department of Epidemiology and Preventive Oncology, Alberta Cancer Board, Calgary, Alberta, Canada;* [3]*Department of Pharmacology and Therapeutics, University of Calgary, Calgary, Alberta T2N 4N1, Canada.*

**Summary** Matrix metalloproteinases (MMPs) and their inhibitors (tissue inhibitors of metalloproteinases, TIMPs) play essential roles in the remodelling of the extracellular matrix (ECM). Results of *in vivo* and *in vitro* studies suggest that the balance between MMPs and TIMPs is altered in neoplasia, contributing to the invasive and metastatic properties of malignant tumours. In this study we have analysed the expression of five MMP genes and TIMP-1 and TIMP-2 in 37 benign and malignant lesions of human breast using Northern blot analysis. MMP-9 (92 kDa gelatinase) and MMP-11 (stromelysin 3) were most consistently expressed by carcinomas. Based on detection of either MMP-9 or MMP-11 mRNAs, we were able to distinguish between malignant and benign disease with a predictive accuracy of 90% with 94% sensitivity and 85% specificity. Subsequently, these results were compared with results for carcinomas of colon and lung and malignant non-Hodgkin's lymphomas (NHL). Elevated MMP-9 and TIMP-1 expression was observed in all four systems. MMP-11 characterised all carcinomas as well as carcinomas *in situ* but was not detectable in NHL. Our data therefore argue that there are remarkably similar patterns of specific functions involved in ECM remodelling that correlate with malignancy in different human tumours of different histogenesis. However, MMP-11 expression is a characteristic of tumours of epithelial origin that is not found in lymphoid neoplasia. Thus it suggests that MMP-11 may play a regulatory role in the invasion and metastasis of carcinomas.

**Keywords:** breast neoplasia; extracellular matrix; metalloproteinases; tissue inhibitors of metalloproteinases

The ability to breach tissue boundaries through active destruction of extracellular matrix (ECM) is a *sine qua non* of tumour invasion, dissemination and metastasis formation (Liotta *et al.*, 1991). It is, in fact, the explicit definition of malignancy. It is therefore axiomatic that the events involved in tumour-mediated ECM dissolution can help to provide an understanding of tumour progression at the molecular level.

Over the past two decades, there has been an explosion of information about the molecular processes involved in ECM breakdown (reviewed in Liotta *et al.*, 1991; Mignatti *et al.*, 1986; Mignatti and Rifkin, 1993; Stetler-Stevenson *et al.*, 1993; Alexander and Werb, 1991). The picture of normal ECM remodelling that has emerged is of a highly regulated process involving: (1) multiple secreted proteinases from different families; (2) the controlled local activation of these enzymes; and (3) the actions of specific proteinase inhibitors. The family of zinc-dependent matrix metalloproteinases (MMPs) that includes collagenases, stromelysins and gelatinases (also known as type IV collagenases) has achieved pre-eminence since *in vitro* work has shown that, in many situations, they are the principal players responsible for the actual attack on ECM components (Alexander and Werb, 1991; Mignatti *et al.*, 1986). In particular, several family members, including the 72 kDa and 92 kDa type IV gelatinases A and B (MMP-2 and MMP-9) can degrade basement membrane components and have been shown to be instrumental in tumour invasion *in vitro* (Liotta *et al.*, 1991; Mignatti and Rifkin, 1993; Stetler-Stevenson *et al.*, 1993; Collier *et al.*, 1988; Wilhelm *et al.*, 1989; Nakajima *et al.*, 1987; Watanabe *et al.*, 1993; Juarez *et al.*, 1993). Moreover, deregulation of the balance between MMPs and their inhibitors, the tissue inhibitors of metalloproteinases (TIMPs) has been linked to invasive and metastatic behaviour (Liotta *et al.*, 1991; Albini *et al.*, 1991; Khokha *et al.*, 1989, 1992; DeClerck *et al.*, 1991, 1992).

Several pieces of evidence suggest that MMP/TIMP imbalance may be a significant factor in the pathophysiology of mammary cancer. Work with rodent mammary carcinoma cell lines has shown that metastatic ability correlates with increased type IV collagenase activity (Nakajima *et al.*, 1987) and decreased TIMP expression (Korczak *et al.*, 1991). Most importantly, a novel member of the MMP family, designated stromelysin 3 (MMP-11), was recently isolated on the basis of its elevated expression in stromal cells surrounding invasive human breast carcinomas (Basset *et al.*, 1990; Wolf *et al.*, 1993). Moreover, Zucker *et al.* (1993) have found evidence for elevated levels of MMP-9 circulating in plasma of breast cancer patients. Other data from *in situ* hybridisation and immunohistochemical studies have implicated MMP-2 in the invasiveness of mammary carcinomas (Monteagudo *et al.*, 1990; Poulsom *et al.*, 1993; Clavel *et al.*, 1992).

While powerful insights can be provided by studies of transformed or tumour-derived cells *in vitro*, it is necessary to examine fresh human tumours to understand the contributions made by individual MMPs and TIMPs and other ECM remodelling proteins to malignant behaviour. We have previously carried out systematic studies of the expression at the RNA level of MMPs and TIMPs in human sporadic colorectal neoplasia (HSCN) (Urbanski *et al.*, 1993), lung carcinomas (Urbanski *et al.*, 1992) and malignant non-Hodgkin's lymphomas (NHL) (Kossakowska *et al.*, 1991, 1992, 1993). In one of these studies we showed, using statistical analysis, that the level of expression of MMP-9 transcripts correlated well with the clinical aggressiveness of immunoblastic NHL, strongly implicating this enzyme in dissemination of these tumours (Kossakowska *et al.*, 1992).

In the present work, we turn our attention to human breast cancer, carrying out a systematic assessment of the expression at the RNA level of five members of the MMP family, namely interstitial collagenase (MMP-1), MMP-2, matrilysin (MMP-7), MMP-9 and MMP-11, along with

Correspondence: AE Kossakowska, Department of Pathology, Foothills Hospital, 1403-29 St. NW, Calgary, Alberta, T2N 2T9, Canada
Received 10 July 1995; revised 7 December 1995; accepted 3 January 1996

TIMP-1 and TIMP-2. Expression patterns are assessed statistically for relationships with the morphological characteristics of 37 breast lesions.

### Materials and methods

### *Tissue collection and preparation*

All tissue was received fresh from the operating room. Analysed cases included 17 carcinomas (12 infiltrating duct, two lobular, two medullary and one mucinous), two intraductal carcinomas, one large intraductal papilloma, five fibroadenomas, three cases of benign non-proliferative breast disease, seven samples of breast tissue adjacent to either fibroadenoma (one case) or carcinoma (three showing benign proliferative breast disease without atypia and three showing non-proliferative breast disease) and two metastatic breast carcinomas (one to skin and one to axillary lymph node). The tissue submitted for RNA extraction was embedded in OCT. Cryostat sections taken adjacent to the analysed tissue and stained with haematoxylin and eosin precisely identified the analysed lesions.

### *Histological assessment*

All breast pathology was assessed independently by two pathologists. Diagnoses and terminology were those in current use in the surgical pathology division of the Department of Pathology, University of Calgary. Sections taken adjacent to the tissue used for RNA extraction were assessed for degree of desmoplasia in epithelial neoplastic lesions and for stromal proliferation in fibroadenomas and non-neoplastic breast (none, –; mild, +; moderate, + +; severe, + + +) in order to assess stromal activity and its correlation with MMP-11 expression.

### *RNA extraction and Northern blot analysis*

Total cellular RNA from tissue samples was extracted by the acid – guanidinium – thiocyanate – phenol – chloroform (AGPC) method (Chomczynski and Sacchi, 1987). Frozen tissue was homogenised in guanidinium isothiocyanate solution and subsequently extracted with acid phenol–chloroform. The precipitated RNA was washed, ethanol-precipitated, resuspended in 0.5% sodium dodecyl sulphate (SDS) solution and stored at −70°C. Northern blot analysis was performed according to previously described methodology (Sambrook *et al.*, 1989; Urbanski *et al.*, 1992). Total cellular RNA (10 $\mu$g) was applied to each well and electrophoresed through formaldehyde-containing agarose gels. The products of electrophoresis were transferred onto Duralon membranes and fixed with a UV cross-linker. Six identical blots containing the same amount of each sample were prepared simultaneously and hybridised with $^{32}P$ nick-translated DNA probes in standard 50% formamide-containing hybridisation buffer (Sambrook *et al.*, 1989). The blots were washed, autoradiographed and subsequently stripped of the labelled probe for reuse by incubation with prehybridisation solution for 15 min at 80°C. Autoradiographic signals were quantified using a Pharmacia UltroScan XL laser densitometer, and all signals were within the linear response range of the X-ray film and the detection system. Each blot carried a control sample of RNA derived from Hs68 human foreskin fibroblasts that had been stimulated for 8 h with $10^{-7}$ M phorbol myristoyl-13 acetate. Signals were normalised between different blots based on the signals from the Hs68 RNA control. Signals were expressed as strong (+ + +, corresponding to normalised densitometric measurements greater than 3.0 absorbance units), moderate (+ +, densitometry measurements between 1.0 and 2.9) or weak (+, densitometry measurements less than 1.0). Samples were loaded on the gels in coded fashion and the blots were interpreted without knowledge of the tissue type.

### *DNA probes*

The human TIMP-1, TIMP-2, MMP-1, MMP-2, MMP-7, MMP-9 and MMP-11 DNA probes have been described previously (Urbanski *et al.*, 1992, 1993; Kossakowska *et al.*, 1991, 1992, 1993).

### *Statistical analysis*

The relationship between the malignant phenotype and MMP and TIMP mRNA values was assessed using the chi-square statistic (Norusis, 1988). The potential of MMP and TIMP mRNA measurements to discriminate between tumours with and without the malignant phenotype was assessed by calculating their sensitivity, specificity and overall predictive accuracy (Rimm *et al.*, 1980). Sensitivity measures the proportion of those tumours with malignant phenotype that are identified by the expression of the mRNA transcripts. Specificity measures the proportion of tumours without the malignant phenotype which are correctly identified by their absence of mRNA transcripts. Predictive accuracy is the proportion of all tumours correctly identified as either malignant or benign by whether they express the mRNA transcripts.

The specimens were further subdivided into seven categories of neoplasia. Kendall's tau was the statistic (Norusis, 1988; Rimm *et al.*, 1980) used to test the association between mRNA expression and degree of neoplasia.

### Results

The human breast tissue specimens ranged along the continuum of neoplasia from morphologically benign ($n=16$) and preinvasive epithelial lesions ($n=2$) through malignant ($n=17$) to metastatic ($n=2$). Figure 1 shows representative Northern blot results for 13 samples of human breast. Levels of expression of MMP and TIMP transcripts were assessed as strong (+ + +), moderate (+ +), weak (+) or absent (−) by visual comparison with signals obtained from human fibroblast RNA that was included as a control on each blot. The full results of this analysis are shown in Tables I and II.

It can be seen that the genes were expressed to varying

**Figure 1** Representative results of the Northern blot analysis of non-neoplastic and neoplastic breast lesions probed for MMP-9, MMP-11, TIMP-1 and TIMP-2. F, fibroblasts; FA, fibroadenoma, N*, non-neoplastic breast adjacent to fibroadenoma; CIS, carcinoma *in situ*; ID Ca, infiltrating duct carcinoma; N, benign non-proliferative breast disease; $N^+$, non-neoplastic breast adjacent to carcinoma; MET, metastasis).

**Table I** Summary of expression of MMP and TIMP RNAs in malignant breast tissues

| | *MMP-1* | *MMP-2* | *MMP-7* | *MMP-9* | *MMP-11* | *Desmoplasia* | *TIMP-1* | *TIMP-2* L | S |
|---|---|---|---|---|---|---|---|---|---|
| Ductal | | | | | | | | | |
| 1 | NA | NA | NA | – | NA | NA | ++ | NA | NA |
| 2 | – | – | – | + | + | + | +++ | + | + |
| 3 | + | + | – | – | + | + | +++ | + | + |
| 4 | – | – | + | + | ++ | ++ | +++ | + | + |
| 5 | + | + | – | – | + | + | +++ | + | + |
| 6 | – | – | – | + | +++ | ++ | +++ | ++ | + |
| 7 | – | – | – | – | + | + | + | + | + |
| 8 | NA | NA | NA | NA | + | + | – | + | + |
| 9 | – | – | – | – | + | 0 | + | NA | NA |
| 10 | – | – | – | + | ++ | ++ | ++ | + | + |
| 11 | – | – | – | + | ++ | ++ | ++ | + | + |
| 12 | NA | NA | + | + | + | + | ++ | + | + |
| Lobular | | | | | | | | | |
| 1 | NA | NA | NA | NA | + | + | ++ | + | + |
| 2 | NA | NA | – | – | ++ | + | +++ | + | + |
| *In situ* | | | | | | | | | |
| 1 | – | ++ | – | + | + | + | +++ | ++ | ++ |
| 2 | – | – | – | – | – | 0 | + | NA | NA |
| Miscellaneous | | | | | | | | | |
| 1 | – | – | – | + | ++ | + | +++ | + | + |
| 2 | – | – | – | + | – | 0 | ++ | NA | NA |
| 3 | – | – | ++ | + | + | + | ++ | + | + |
| Metastasis | | | | | | | | | |
| 1 | + | + | – | + | – | 0 | +++ | + | ++ |
| 2 | + | – | – | + | – | 0 | ++ | – | – |

NA, not available.

**Table II** Summary of expression of MMP and TIMP RNAs in benign breast tissues

| | *MMP-1* | *MMP-2* | *MMP-7* | *MMP-9* | *MMP-11* | *Stromal proliferation* | *TIMP-1* | *TIMP-2* L | S |
|---|---|---|---|---|---|---|---|---|---|
| *Adjacent to FA* | | | | | | | | | |
| 1 | + | – | + | – | – | – | ++ | + | + |
| *Benign non-proliferative breast disease* | | | | | | | | | |
| 1 | – | – | NA | NA | – | – | ++ | – | – |
| 2 | + | + | + | – | – | – | + | + | + |
| 3 | + | + | + | – | – | – | ++ | + | + |
| *Adjacent to carcinoma* | | | | | | | | | |
| 1 | – | + | – | – | – | – | + | + | + |
| 2 | + | + | – | – | + | + | +++ | + | + |
| 3 | NA | NA | NA | NA | – | – | + | + | + |
| 4 | – | – | – | – | – | – | + | NA | NA |
| 5 | – | – | – | – | – | – | + | + | + |
| 6 | – | – | – | – | – | – | + | NA | NA |
| *Fibroadenomas* | | | | | | | | | |
| 1 | + | + | + | – | – | + | +++ | + | + |
| 2 | – | – | + | – | – | + | ++ | + | + |
| 3 | NA | NA | NA | NA | + | + | + | + | + |
| 4 | – | – | – | – | – | + | + | NA | NA |
| 5 | – | – | – | + | – | + | ++ | NA | NA |
| *Papilloma with moderate dysplasia* | | | | | | | | | |
| 1 | – | – | – | – | – | – | + | NA | NA |

NA, not available.

degrees: TIMP-1 and TIMP-2 transcripts were present in almost all specimens, MMP-11 and MMP-9 in about one-half, and only one-quarter had detectable MMP-1, MMP-2 and MMP-7 RNAs. Secondly, mRNAs encoding MMP-1, MMP-2, MMP-7 and TIMP-2 appeared unrelated to the malignant phenotype, since they were expressed in both benign and malignant lesions.

The third and most important point from this study comes from statistical analysis of the expression of MMP-11, MMP-9 and TIMP-1. In the 31 specimens for which results were available for all three, MMP-11 distinguished malignant disease with the greatest predictive accuracy (84% *vs* 77% and 71% respectively). Fourteen of the 18 malignant specimens and 12 of the 13 benign were correctly identified by the presence or absence, respectively, of transcripts for MMP-11. This yielded a predictive accuracy of 84% [(14+12)/(18+13)]. However, as shown in Figure 2, MMP-9 was the most strongly related to phenotype among the benign ($n=13$), malignant ($n=16$) and metastatic ($n=2$) categories ($P=0.0004$) and along a seven category continuum ($P=0.0003$). This was likely because MMP-11 transcripts were not expressed in the two specimens of metastatic disease (Tables I and II). Detection of either MMP-11 or MMP-9 increased the predictive accuracy to 90% with 94% sensitivity and 85% specificity. Eleven of the 13 benign specimens expressed neither MMP-11 nor MMP-9, while 17 of the 18 malignant specimens expressed either or both of MMP-11 and MMP-9 [(11+17)/(13+18)=90% predictive accuracy] (Table III).

Specimens that deviated from the pattern of nonexpression of MMP-11 and MMP-9 in benign tissues and expression in malignant tissues probably represent transitional stages on the progression continuum. Thus, the one malignant primary tumour that expressed neither MMP-11 or MMP-9 was carcinoma *in situ*. Two of the 13 benign tumours expressed either MMP-11 or MMP-9; these were a fibroadenoma expressing MMP-9 and benign tissue adjacent to a carcinoma and expressing MMP-11.

We also observed that some MMP and TIMP genes showed differential expression at the periphery of the tumours compared with samples from the centre (Table IV). Transcripts for MMP-11 and TIMP-1 were elevated in the periphery of two of the five tumours analysed in this way, though only one case showed elevation of both transcripts. Also, in one case we found increased MMP-7 RNA levels in the periphery. Such patterns were not seen for MMP-1, MMP-2, MMP-9 or TIMP-2. Histological examination of sections adjacent to areas of tumour taken for RNA extraction were assessed for degree of desmoplasia as an indicator of stromal activity. As can be seen from Tables I and II, degree of desmoplasia was fairly well correlated with MMP-11 expression.

The comparison of the expression patterns of MMPs across four tumour systems is summarised in Figure 3. The most striking pattern is the association of MMP-11 expression with all types of carcinoma that we have analysed, and the absence of MMP-11 transcripts in NHL. Regardless of the type of malignancy, TIMPs 1 and 2 were expressed by over 80% of malignant lesions and MMP-9 by at least 50%. The expression of MMP-2, MMP-1 and MMP-7 showed the greatest variability across types of malignancy. But consistent features of all tumour types are the expression of MMP-9 or overexpression of TIMP-1.

**Figure 2** MMP mRNA expression (percentage of samples) in breast tissue by neoplastic phenotype. The breast tissue specimens were arranged in a seven category continuum of increasing malignancy (from left to right). The figure shows the percentage of samples in each category that expressed detectable transcripts of MMP-9 and MMP-11. MMP-9 was most strongly related to degree of neoplasia, whereas MMP-11 most clearly distinguished the neoplastic phenotype from the benign. □, MMP-9; ■, MMP-11.

### Discussion

The natural history of malignant tumours involves multiple episodes of ECM breakdown mediated by secreted ECM-degrading proteinases (Liotta *et al.*, 1991; Mignatti and Rifkin, 1993). A comprehensive picture of the molecular machinery that can participate in matrix turnover has emerged from *in vitro* studies of degradative enzymes and their natural inhibitors (Mignatti and Rifkin, 1993; Stetler-Stevenson *et al.*, 1993; Alexander and Werb, 1991). However, most of the current literature that deals with the roles of proteinases in tumour invasion and metastasis has come from studies of transformed or tumour-derived cell lines in culture. To fully appreciate their biological significance, it is necessary to study factors that have been implicated in ECM destruction in the natural context of the complex tumour–host interactions in freshly resected human tumours. The members of the MMP and TIMP gene families are the focus of this work.

**Table III** Frequency of mRNA transcript expression for MMP-9 and MMP-11 by tissue type ($n$=31)

| *Tissue type* (n) | *MMP-9 only* | *Both MMP-9 and MMP-11* | *MMP-11 only* | *Total (MMP-11 and MMP-9)* n | % |
|---|---|---|---|---|---|
| Benign (13) | 1 | 0 | 1 | 2 | 15 |
| Malignant primary (16) | 1 | 9 | 5 | 15 | 94 |
| Metastatic (2) | 2 | 0 | 0 | 2 | 100 |

**Table IV** Expression patterns of mRNA transcripts of MMPs and TIMPs in the central and peripheral samples from breast carcinomas ($n$=5)

| *Location* | *MMP-1* | *MMP-2* | *MMP-7* | *MMP-9* | *MMP-11* | *TIMP-1* | *L* | *S* |
|---|---|---|---|---|---|---|---|---|
| Centre | – | – | + | + | + + | + + | + | + |
| Periphery | – | – | NA | NA | + + | + + + | + | + |
| Centre | – | – | – | – | + | + + | + | + |
| Periphery | – | – | – | + | + + + | + + | NA | NA |
| Periphery | NA | NA | NA | NA | + + + | + + + | + + | + |
| Centre | NA | NA | – | + | NA | + + | + | + |
| Periphery | – | – | + + | + | + | + + | NA | NA |
| Centre | – | – | – | – | – | + | + | + |
| Periphery | – | – | – | – | + | + | + | + |
| Centre | – | – | – | + | – | + + | NA | NA |
| Periphery | – | – | – | + | – | + + | NA | NA |

NA, not available.

**Figure 3** Comparison of MMP mRNA expression (percentage of samples) in malignant lesions of lung, colon, breast and lymphoid tissue. The figure compares the results obtained here for expression of MMP-1 ( ), MMP-2 ( ), MMP-7 ( ), MMP-9 ( ) and MMP-11 ( ) in malignant breast lesions with our results for malignant lesions of lung (Urbanski *et al.*, 1992) and colon (Urbanski *et al.*, 1993) and non-Hodgkin's lymphomas (NHL, Kossakowska *et al.*, 1991, 1992, 1993). MMP-11 was expressed in almost all malignant lesions of epithelial origin but was absent in NHL. MMP-9 was expressed in most malignant lesions, whereas MMP-1, MMP-2 and MMP-7 varied considerably across tumour systems. *Includes three metastatic (one lung, two breast) two *in situ* (breast) lesions.

This study reports a thorough analysis of expression at the RNA level of MMP-1, -2, -7, -9 and -11 and TIMP-1 and -2 in a spectrum of breast tissues ranging from normal to overtly malignant. Comparative assessment of expression levels alongside pathological characteristics of the lesions allows us to identify which of these genes are likely important correlates of the malignant phenotype. Further, to explore the generality of the associations at which we arrive, we have contrasted the data obtained from human breast carcinomas with those from other tumours of both epithelial (lung and colon carcinomas) and non-epithelial origin (non-Hodgkin's lymphomas; NHL).

A number of caveats to the present study must be acknowledged. First, we recognise that other enzymes of the aspartyl, cysteine and serine proteinase families and their specific inhibitors are also likely to be significant participants in ECM remodelling during cancer progression. Second, we have limited our analyses to mRNA detection since it provides the simplest and most reliable method of quantification of expression for the functions involved. Since MMPs and TIMPs are for the most part secreted freely diffusible proteins, both quantification at the protein level and immunohistochemical localisation present significant problems (Clavel *et al.*, 1992; Hembry *et al.*, 1986). Although it is possible that mRNA levels for MMPs and TIMPs may not always reflect amounts of protein products secreted, as has been reported for MMP-1 in a rat epithelial cell line (Whitelock *et al.*, 1993), RNA analyses provide a generally reliable means of assessment of expression. Third, it is clear that the activities of MMPs are controlled at multiple levels including expression, activation and via interaction with TIMPs. Thus expression and secretion of protein product may not, in all cases, be the chief arbiters of functionality (Azzam *et al.*, 1993). For instance, activation of pro-72 kDa gelatinase A (MMP-2) involves binding of the pro-MMP2/TIMP-2 complex to a cell-surface receptor followed by proteolytic cleavage by a transmembrane metalloproteinase (MT-MMP-1), thereby restricting MMP-2 activity to the pericellular environment of cells carrying appropriate activation machinery (Emonard *et al.*, 1992; Monsky *et al.*, 1993; Kleiner and Stetler-Stevenson, 1993; Sato *et al.*, 1994; Vassalli and Pepper, 1994). Finally, there is the possibility that the expression of genes that are important in ECM destruction may be spatially restricted to the invasion front of the tumour (Stetler-Stevenson *et al.*, 1993). This may complicate quantification owing to variable tissue sampling and because the expressing cells may be vastly outnumbered by non-expressing cells. Because of these concerns, it is important to emphasise that while our data identify two MMP genes by virtue of the association of their expression with malignancy, we cannot exclude the possibility that others may also play significant roles.

From our studies it is apparent that expression of MMP-11 and MMP-9 is likely associated with malignant phenotype in human breast carcinomas. Using detection of either MMP-11 or MMP-9 transcripts as a criterion, we were able to distinguish malignant from normal tissue with a predictive accuracy of 90% with 94% sensitivity and 85% specificity. Moreover, these genes are consistently found to be expressed in carcinomas in three different tissues of origin. As in HSCN (Urbanski *et al.*, 1993), preinvasive lesions of human breast are intermediate in their expression patterns of MMPs and TIMPs between normal and frankly malignant tissue. However, the presence of MMP-11 and MMP-9 transcripts is not simply related to epithelial proliferation, as one case of large papilloma (breast) with atypia showed neither MMP-11 nor MMP-9 expression. Similar observations were made previously in HSCN where many colonic adenomas did not show altered MMP expression (Urbanski *et al.*, 1993). Thus, the processes that control cell proliferation and invasion are to some degree, independent. This conclusion differs from that of a recent *in vitro* study of human A431 epidermoid carcinoma cells, in which MMP-9 expression was observed in sparse, proliferating cultures but not in dense, contact-inhibited ones (Xie *et al.*, 1994). The results with cultured A431 cells may reflect the behaviour of malignant cells in a highly advanced tumour *in vivo*, in which cells at the invasion front are more metabolically active than cells in the centre of the tumour, and thus also express MMP-9 to the highest level. However, our data clearly show that in benign proliferative breast lesions *in vivo*, MMP-9 expression and epithelial proliferation are uncoupled. We suggest that altered expression of both MMP-9 and MMP-11 characterises epithelial lesions committed to malignant transformation and may reflect underlying alterations in regulatory tumour-suppressor genes or proto-oncogenes that prepare the cells for imminent invasion.

Several reports localised MMP-11 to the invasive front in breast carcinomas where it is expressed not by tumour cells but by stromal cells (Basset *et al.*, 1990; Wolf *et al.*, 1993). Similar findings have been reported for basal cell carcinoma (Wagner *et al.*, 1992) and head and neck squamous cell carcinomas (Muller *et al.*, 1993). Support for the notion that tumour–stromal interactions are important for setting up MMP-11 expression comes from our observations that expression of MMP-11 transcripts was highest in samples taken from the tumour periphery in two out of five cases, and that MMP-11 expression was also detectable in tissue immediately adjacent to a carcinoma. Moreover, degree of desmoplasia correlated well with MMP-11 expression (Tables I and II). It is therefore of considerable interest that we find that MMP-11 expression is restricted to carcinomas and preinvasive lesions but is undetectable in 42 cases of NHL that we have also analysed. As progression of NHL is not associated with destruction of basement membrane it is likely that MMP-11 expression is intimately linked with events that lead to the invasion of tumours of epithelial origin through this biological barrier. However, MMP-11 does not itself display detectable proteolytic activity towards ECM components (G Murphy *et al.*, 1993), though it can cleave and inactivate serine proteinase inhibitor - $\alpha_1$-proteinase inhibitor (Pei *et al.*, 1994). Thus, it is possible that it may perform a regulatory role by controlling the activities of other proteinases. Wolf *et al.* (1993) have noted similarities in the expression patterns of MMP-11 and urokinase plasminogen activator in breast carcinomas, suggesting that both enzymes may cooperate during cancer progression.

Although we found MMP-11 expression in 84% of primary breast carcinomas, we failed to detect it in the skin and nodal metastases. These findings do not agree with those of Wolf *et al.* (1993) who observed MMP-11 in 11 out of 13 (85%) lymph node metastases of breast carcinoma. We note however, that these authors report lower levels of MMP-11 detection in metastases to other locations (67% to bone and 50% to other organs). If MMP-11 is indeed linked to invasive spread of tumour cells away from primary carcinomas we think it reasonable, particularly in the light of our inability to detect MMP-11 in NHL, that the different stromal environment of nodal metastases will probably be reflected in the elaboration of different ECM degradative functions, which may also vary from site to site. Clearly, the role of MMP-11 in the growth of metastases is a topic for further investigation using a larger series of tumour specimens. MMP-9 is the other consistent marker of malignancy that is expressed at elevated levels, not only in breast cancer but also in the other three types of malignancy that we have compared. Previously, we showed that MMP-9 expression in a subset of malignant high-grade NHL correlated with clinical aggressiveness as judged by poorer survival (Kossakowska *et al.*, 1992). Our findings are supported by the demonstration of elevated levels of MMP-9 protein in the plasma of patients with colon and breast cancer (Zucker *et al.*, 1993). In a zymographic analysis of bladder cancer specimens, Davies *et al.* (1993) have correlated increased amounts of MMP-9 and active MMP-2 with tumour grade and invasion. The links between MMP-9 and cellular invasion are now very strong and include work with oncogenically transformed rat fibroblasts (Bernhard *et al.*, 1992), oral squamous cell carcinoma-derived cell lines (Juarez *et al.*, 1993), U937 monoblastoid cells (Watanabe *et al.*, 1993) as well as non-neoplastic cell types capable of invasion, such as preimplantation mouse and human blastocysts (Behrendtsen *et al.*, 1992; Librach *et al.*, 1991). Unlike the predominantly stromal expression of MMP-11 and MMP-2 transcripts (Basset *et al.*, 1990, 1994; Wolf *et al.*, 1993; Polette *et al.*, 1994; Soini *et al.*, 1994) MMP-9 RNAs are localised primarily in epithelial cells of breast carcinoma (Soini *et al.*, 1994). This indicates that the increased expression of MMP-9 and MMP-11 seen in malignant tumours involves distinct mechanisms.

Although our data point to an association of both MMP-9 and MMP-11 with the malignant phenotype in breast cancer, the situation with MMP-1, MMP-2 and MMP-7 is less clear. Transcripts for the last genes were not consistently found in malignant tumours and were frequently detected in benign lesions or normal tissues adjacent to cancer. However, as we pointed out earlier, the absence of association between gene expression and malignancy does not indicate non-involvement of the products of these genes in the malignant process. In fact, our data indicate an association of MMP-1 with the malignant phenotype in colon and lung carcinomas but not in breast carcinoma or NHL. Its involvement may thus depend on the anatomical location of the tumour. In colonic neoplasia, MMP-1 transcripts are localised to eosinophils, fibroblasts and endothelial cells (Gray *et al.*, 1993), again demonstrating the importance of tumour–host interactions.

Our data on TIMP-1 and TIMP-2 reveal that transcripts for both are present in most tissues whether benign or malignant. Consistently we have seen an elevation of TIMP-1 expression in all types of malignant lesions studied, and we previously showed localisation of TIMP-1 transcripts to stromal cells in NHL (Kossakowska *et al.*, 1991) and lung carcinomas (Urbanski *et al.*, 1992). TIMP-1 transcription is highly inducible *in vitro* by diverse cytokines whereas TIMP-2 is expressed in a more constitutive fashion (Leco *et al.*, 1992; Stetler-Stevenson *et al.*, 1990). So tumour–host interactions would again appear to be important for the selective up-regulation of TIMP-1 expression. We do not yet know the full significance of increased TIMP-1 production on tumour pathophysiology. It could be interpreted as an attempt by the host to limit the ECM remodelling that is elicited by the presence of the tumour cells. However, as both TIMP-1 and TIMP-2 have growth modulatory actions, in addition to their roles as MMP inhibitors (Bertaux *et al.*, 1991; Hayakawa *et al.*, 1992; AN Murphy, *et al.*, 1993; Nemeth and Goolsby, 1993), it is possible that increased TIMP-1 production at the tumour–host interface may in certain situations stimulate tumour growth rather than having the expected effect of limiting cellular invasion.

In summary, the observations presented here confirm a strong association between expression of MMP-11 mRNA and malignant phenotype in human breast epithelial neoplasms, and for the first time, indicate that MMP-9 is also consistently overexpressed in these carcinomas. This subset of MMPs is further implicated as a factor common to the malignant process in carcinomas of other organs, but we find no evidence for involvement of MMP-11 in NHL, which suggests that this proteinase characterises progression of carcinomas. The proteinases that appear to be necessary for invasion can be expressed in preinvasive states, indicating their potential applications as prognostic indices and as targets for therapeutic intervention. Further research into the molecular mechanisms underlying the expression of MMP-11 and MMP-9 may permit manipulation of the neoplastic process to inhibit or prevent acquisition of the invasive phenotype.

**Acknowledgements**
This work was supported by a grant from the Foothills Hospital Research and Development Fund to AEK (project no. 955) and by grants from the Medical Research Council of Canada to DRE (MT-10572) and AEK (MT-12706). DRE is an Alberta Heritage Foundation for Medical Research Senior Scholar. We are grateful to Miss Tracy Allen and Ms Nona Bissillion for secretarial assistance.

### References

ALBINI A, MELCHIORI A, SANTI L, LIOTTA LA, BROWN PD AND STETLER-STEVENSON WG. (1991). Tumour cell invasion inhibited by TIMP-2. *J. Natl Cancer Inst.*, **83,** 775–779.

ALEXANDER CM AND WERB Z. (1991). Extracellular matrix degradation. In *Cell Biology of Extracellular Matrix*. Hay ED, (ed.) pp. 255–302. Plenum Press: New York.

AZZAM HS, ARAND G, LIPPMAN ME AND THOMPSON EW. (1993). Association of MMP-2 activation potential with metastatic progression in human breast cancer cell lines independent of MMP-2 production. *J. Natl Cancer Inst.*, **85,** 1758–1765..

BASSET P, BELLOCQ JP, WOLF C, STOLL I, HUTIN P, LIMACHER JM, PODHAJCER OL, CHENARD MP, RIO MC AND CHAMBON P. (1990). A novel metalloproteinase gene specifically expressed in stromal cells of breast carcinomas. *Nature*, **348,** 699–704.

BASSET P, WOLF C, ROUYER N, BELLOCQ J-P, RIO M-C AND CHAMBON P. (1994). Stromelysin-3 in stromal tissue as a control factor in breast cancer behavior. *Cancer*, **74,** 1045–1049.

BEHRENDTSEN O, ALEXANDER CM AND WERB Z. (1992). Metalloproteinases mediate extracellular matrix degradation by cells from mouse blastocyst outgrowths. *Development*, **114,** 447–456.

BERNHARD EJ, GRUBER SB AND MUSCHEL RJ. (1992). Direct evidence linking expression of matrix metalloproteinase-9 (92-kDa gelatinase/collagenase) to the metastatic phenotype in transformed rat embryo cells. *Proc. Natl Acad. Sci. USA*, **91,** 4293–4297.

BERTAUX B, HORNEBECK W, EISEN AZ AND DUBERTRET L. (1991). Growth stimulation of human keratinocytes by tissue inhibitor of metalloproteinases. *J. Invest. Derm.*, **97**, 679-685.

CHOMCZYNSKI P AND SACCHI N. (1987). Single-step method of RNA isolation by acid guanidinium thiocyanate-phenol-chloroform extraction. *Anal. Biochem.*, **162**, 156-159.

CLAVEL C, POLETTE M, DOCO M, BINNINGER I AND BIREMBAUT P. (1992). Immunolocalization of matrix metalloproteinases and their tissue inhibitor in human mammary pathology. *Bull. Cancer*, **79**, 261-270.

COLLIER IE, WILHELM SM, EISEN AZ, MARMER BL, GRANT GA, SELTZER JL, KRONBERGER A, HE C, BAUER EA AND GOLDBERG GI. (1988). H-ras oncogene-transformed human bronchial epithelial cells (TBE-1) secrete a single metalloproteinase capable of degrading basement membrane collage. *J. Biol. Chem.*, **263**, 6579-6587.

DAVIES B, WAXMAN J, WASAN H, ABEL P, WILLIAMS G, KRAUSZ T, NEAL D, THOMAS D, HANBY A AND BALKWILL F. (1993). Levels of matrix metalloproteinases in bladder cancer correlate with tumour grade and invasion. *Cancer Res.*, **53**, 5365-5369.

DECLERCK YA, YEAN T-D, CHAN D, SHIMADA H AND LANGLEY KE. (1991). Inhibition of tumour invasion of smooth muscle cell layers by recombinant human metalloproteinase inhibitor. *Cancer Res.*, **51**, 2151-2157.

DECLERCK YA, PEREZ N, SHIMADA H, BOONE TC, LANGLEY KE AND TAYLOR SM. (1992). Inhibition of invasion and metastasis in cells transfected with an inhibitor of metalloproteinases. *Cancer Res.*, **52**, 701-708.

EMONARD HP, REMACLE AG, NOEL AC, GRIMAUD J-A, STETLER-STEVENSON WG AND FOIDART J-M. (1992). Tumour cell surface-associated binding site for the $M_r$ 72,000 type IV collagenase. *Cancer Res.*, **52**, 5845-5848.

GRAY ST, YUN K, MOTOORI T AND KUYS YM. (1993). Interstitial collagenase gene expression in colonic neoplasia. *Am. J. Pathol.*, **143**, 663-671.

HAYAKAWA T, YAMASHITA K, UCHIJIMA E AND IWATA K. (1992). Growth-promoting activity of tissue inhibitor of metalloproteinases-1 (TIMP-1) for a wide range of cells. *FEBS Lett*, **289**, 29-32.

HEMBRY RM, MURPHY G, CAWSTON TE, DINGLE JT AND REYNOLDS JJ. (1986). Characterization of a specific antiserum for mammalian collagenase from several species: immunolocalization of collagenase in rabbit chondrocytes and uterus. *J. Cell Sci.*, **81**, 105-123.

JUAREZ J, CLAYMAN G, NAKAJIMA M, TANABE KK, SAYA H, NICOLSON GL AND BOYD D. (1993). Role and regulation of expression of 92-kDa type-IV collagenase (MMP-9) in 2 invasive squamous-cell-carcinoma cell lines of the oral cavity. *Int. J. Cancer*, **55**, 10-18.

KHOKHA R, WATERHOUSE P, YAGEL S, LALA PK, OVERALL CM, NORTON G AND DENHARDT DT. (1989). Antisense RNA-induced reduction in murine TIMP levels confers oncogenicity on Swiss 3T3 cells. *Science*, **243**, 947-950.

KHOKHA R, ZIMMER MJ, GRAHAM CH, LALA PK AND WATERHOUSE P. (1992). Suppression of invasion by inducible expression of tissue inhibitor of metalloproteinase-1 (TIMP-1) inB16-F10 melanoma cells. *J. Natl Cancer Inst.*, **84**, 1017-1023.

KLEINER DE JR AND STETLER-STEVENSON WG. (1993). Structural biochemistry and activation of matrix metalloproteinases. *Cell Biol.*, **5**, 891-897.

KORCZAK B, KERBEL RS AND DENNIS JW. (1991). Autocrine and paracrine regulation of tissue inhibitor of metalloproteinases, transin, and urokinase gene expression in metastatic and nonmetastatic mammary carcinoma cells. *Cell Growth Differ.*, **2**, 335-341.

KOSSAKOWSKA AE, URBANSKI SJ AND EDWARDS DR. (1991). Tissue inhibitor of metalloproteinases-1 (TIMP-1) RNA is expressed at elevated levels in malignant non-Hodgkin's lymphomas. *Blood*, **77**, 2475-2481.

KOSSAKOWSKA AE, URBANSKI SJ, HUCHCROFT SA AND EDWARDS DR. (1992). Relationship between the clinical aggressiveness of large cell immunoblastic lymphomas and expression of 92 kDa gelatinase (type IV collagenase) and tissue inhibitors of metalloproteinases-1 (TIMP-1) RNAs. *Oncol. Res.*, **4**, 223-240.

KOSSAKOWSKA AE, URBANSKI SJ, WATSON A, HAYDEN LJ AND EDWARDS DR. (1993). Patterns of expression of metalloproteinases and their inhibitors in human malignant lymphomas. *Oncol. Res.*, **5**, 19-28.

LECO KJ, HAYDEN LJ, SHARMA RR, ROCHELEAU H, GREENBERG AH AND EDWARDS DR. (1992). Differential regulation of *TIMP*-1 and *TIMP*-2 mRNA expression in normal and Ha-*ras*-transformed murine fibroblasts. *Gene*, **117**, 209-217.

LIBRACH CL, WERB Z, FITZGERALD ML, CHIU K, CORWIN NM, ESTEVES RA, GROBELNY D, GALARDY R, DAMSKY CH AND FISHER SJ. (1991). 92-kD type IV collagenase mediates invasion of human cytotrophoblasts. *J. Cell Biol.*, **113**, 437-449.

LIOTTA LA, STEEG PS AND STETLER-STEVENSON WG. (1991). Cancer metastasis and angiogenesis: an imbalance of positive and negative regulation. *Cell*, **64**, 327-336.

MIGNATTI P AND RIFKIN DB. (1993). Biology and biochemistry of proteinases in tumour invasion. *Physiol. Rev.*, **73**, 161-195.

MIGNATTI P, ROBBINS E AND RIFKIN DB. (1986). Tumour invasion through the human amniotic membrane: requirement for a proteinase cascade. *Cell*, **47**, 486-498.

MONSKY WL, KELLY T, LIN C-Y, YEH Y, STETLER-STEVENSON WG, MUELLER SC AND CHEN W-T. (1993). Binding and localization of $M_r$ 72,000 matrix metalloproteinase at cell surface invadopodia. *Cancer Res.*, **53**, 3159-3164.

MONTEAGUDO C, MERINO MJ, SAN-JUAN J, LIOTTA LA AND STETLER-STEVENSON WG. (1990). Immunohistochemical distribution of type IV collagenase in normal, benign, and malignant breast tissue. *Ann. J. Pathol.*, **136**, 585-592.

MULLER D, WOLF C, ABECASSIS J, MILLON R, ENGELMANN A, BRONNER G, ROUYER N, RIO M-C, EBER M, METHLIN G, CHAMBON P AND BASSET P. (1993). Increased stromelysin 3 gene expression is associated with increased local invasiveness in head and neck squamous cell carcinomas. *Cancer Res.*, **53**, 165-169.

MURPHY AN, UNSWORTH EJ AND STETLER-STEVENSON WG. (1993). Tissue inhibitor of metalloproteinases-2 inhibits bFGF-induced human microvascular endothelial cell proliferation. *J. Cell. Physiol.*, **157**, 351-358.

MURPHY G, SEGAIN J-P, O'SHEA M, COCKETT M, IOANNOU C, LEFEBVRE O, CHAMBON P AND BASSET P. (1993). The 28-kDa N-terminal domain of mouse stromelysin-3 has the general properties of a weak metalloproteinase. *J. Biol. Chem.*, **268**, 15435-15441.

NAKAJIMA M, WELCH DR, BELLONI PN AND NICOLSON GL. (1987). Degradation of basement membrane type IV collagen and lung subendothelial matrix by rat mammary adenocarcinoma cell clones of differing metastatic potentials. *Cancer Res.*, **47**, 4869-4876.

NEMETH JA AND GOOLSBY CL. (1993). TIMP-2, a growth-stimulatory protein from SV40-transformed human fibroblasts. *Exp. Cell. Res.*, **207**, 376-382.

NORUSIS MJ. (1988). SPSS/PC + V2.0 Base Manual. SPSS Inc: Chicago.

PEI D, MAJMUDAR G AND WEISS SJ. (1944). Hydrolytic inactivation of a breast carcinoma cell-derived serpin by human stromelysin-3. *J. Biol. Chem.*, **269**, 25849-25855.

POLETTE M, GILBERT N, STAS I, NAWROCKI B, NOEL A, REMACLE A, STETLER-STEVENSON WG, BIREMAUT P AND FOIDART J-M. (1994). Gelatinase A expression and localization in human breast cancers. An *in situ* hybridization study and immunohistochemical detection using confocal microscopy. *Virchows Archiv.*, **424**, 641-645.

POULSOM R, HANBY AM, PIGNATELLI M, JEFFERY RE, LONGCROFT JM, ROGERS L AND STAMP GWH. (1993). Expression of gelatinase A and TIMP-2 mRNAs in desmoplastic fibroblasts in both mammary carcinomas and basal cell carcinomas of the skin. *J. Clin. Pathol.*, **46**, 429-436.

RIMM A, HARTZ AJ, KALBFLEISCH JH, ANDERSON AJ AND HOFFMANN RG. (1980). Basic Biostatistics in Medicine and Epidemiology. pp. 56-58. Appleton Century Crofts: New York.

SAMBROOK J, FRITSCH EF AND MANIATIS T. (1989). Molecular Cloning: a Laboratory Manual, 2nd edn. Cold Spring Harbor Laboratory Press: New York.

SATO H, TAKINO T, OKADA Y, CAO J, SHINAGAWA A, YAMAMOTO E AND SEIKI M. (1994). A matrix metalloproteinase expressed on the surface of invasive tumour cells. *Nature*, **370**, 61-65.

SOINI Y, HURSKAINEN T, HOYHTYA M, OIKARINEN A AND AUTIO-HARMAINEN H. (1994). 72 kD and 92 kD type IV collagenase, type IV collagen and laminin mRNAs in breast cancer: a study by *in situ* hybridization. *J. Histochem. Cytochem.*, **42**, 945-951.

STETLER-STEVENSON WG, BROWN PD, ONISTO M, LEVY AT AND LIOTTA LA. (1990). Tissue inhibitor of metalloproteinases-2 (TIMP-2) mRNA expression in tumour cell lines and human tumour tissue. *J. Biol. Chem.*, **265**, 13933-13938.

STETLER-STEVENSON WG, AZNAVOORIAN S AND LIOTTA LA. (1993). Tumour cell interactions with the extracellular matrix during invasion and metastasis. *Cell Biol.*, **9**, 541-573.

URBANSKI SJ, EDWARDS DR, MAITLAND A, LECO KJ, WATSON A AND KOSSAKOWSKA AE. (1992). Expression of metalloproteinases and their inhibitors in primary pulmonary carcinomas. *Br. J. Cancer*, **66**, 1188–1194.

URBANSKI SJ, EDWARDS DR, HERSHFIELD N, HUCHCROFT SA, SHAFFER E, SUTHERLAND L AND KOSSAKOWSKA AE. (1993). Expression pattern of metalloproteinases and their inhibitors changes with the progression of human sporadic colorectal neoplasia. *Diag. Mol. Path.*, **2**, 81–89.

VASSALLI J-D AND PEPPER MS. (1994). Membrane proteases in focus. *Nature*, **370**, 14.

WAGNER SN, RUHRI C, KUNTH K, HOLECEK BU, GOOS M, HOFLER H AND ATKINSON MJ. (1992). Expression of stromelysin 3 in the stromal elements of human basal cell carcinoma. *Diagn. Molec. Pathol.*, **1**, 200–205.

WATANABE H, NAKANISHI I, YAMASHITA K, HAYAKAWA T AND OKADA Y. (1993). Matrix metalloproteinase-9 (92 kDa gelatinase/type IV collagenase) from U937 monoblastoid cells: correlation with cellular invasion. *J. Cell. Sci.*, **104**, 991–999.

WHITELOCK JM, PAINE ML, GIBBINS JR, KEFFORD RF AND O'GRADY RL. (1993). Multiple levels of post-transcriptional regulation of collagenase (matrix metalloproteinase 1) in an epithelial cell line. *Immunol. Cell Biol.*, **71**, 39–47.

WILHELM SM, COLLIER IE, MARMER BL, EISEN AZ, GRANT GA AND GOLDBERG GI. (1989). SV40-transformed human lung fibroblasts secrete a 92-kDa type IV collagenase which is identical to that secreted by normal human macrophages. *J. Biol. Chem.*, **264**, 17213–17221.

WOLF C, ROUYER N, LUTZ Y, ADIDA C, LORIOT M, BELLOCQ J-P, CHAMBON P AND BASSET P. (1993). Stromelysin 3 belongs to a subgroup of proteinases expressed in breast carcinoma fibroblastic cells and possibly implicated in tumour progression. *Proc. Natl Acad. Sci. USA*, **90**, 1843–1847.

XIE B, BUCANA CD AND FIDLER IJ. (1994). Density-dependent induction of 92-kd type IV collagenase activity in cultures of A431 human epidermoid carcinoma cells. *Am. J. Pathol.*, **144**, 1058–1067.

ZUCKER S, LYSIK RM, ZARRABI MH AND MOLL U. (1993). $M_r$ 92,000 type IV collagenase is increased in plasma of patients with colon cancer and breast cancer. *Cancer Res.*, **53**, 140–146.